# Referral gynecological ambulatory clinic: principal diagnosis and distribution in health services

**DOI:** 10.1186/s12905-017-0498-4

**Published:** 2018-01-05

**Authors:** Adna Thaysa Marcial da Silva, Camila Lohmann Menezes, Edige Felipe de Sousa Santos, Paulo Francisco Ramos Margarido, José Maria Soares, Edmund Chada Baracat, Luiz Carlos de Abreu, Isabel Cristina Esposito Sorpreso

**Affiliations:** 1 Division of Gynecology, Medical School, University of São Paulo, São Paulo, SP Brazil; 2 Laboratory of Study Design and Scientific Writing, ABC Medical School, São Paulo, SP Brazil; 3 Avenida Enéas de Carvalho Aguiar, 255 – 10° andar sala 10166, São Paulo, SP CEP: 05403000 Brazil

**Keywords:** Ambulatory care, Health services use, Gynecology, Women’s health, Health care levels

## Abstract

**Background:**

The association between gynecological diagnoses and their distribution in the health sectors provides benefits in the field of women’s health promotion and in medical and interdisciplinary education, along with rationalization according to level of care complexity. Thus, the objective is analyze the clinical-demographic characteristics, main diagnoses in gynecological ambulatory care, and their distribution in health services.

**Method:**

This is a research project of retrospective audit study design with a chart review of data from 428 women treated at University Ambulatory Clinic of Women’s Health, the facility in gynecology and training for Family and Community Medical Residents, São Paulo, Brazil, from 2012 to 2014. Clinical and demographic information, gynecological diagnoses (International Classification of Diseases), and distribution of health services (primary, secondary, and tertiary) were described.

**Results:**

The female patients present non-inflammatory disorders of the female genital tract (81.07%, *n* = 347) and diseases of the urinary system (22.66%, *n* = 97) among the gynecological diagnoses. The chances of having benign breast disease and non-inflammatory disorders of the female genital tract during the reproductive period corresponds to being 3.61 (CI 1.00–16.29) and 2.56 times (CI 1.58–4.16) higher, respectively, than during the non-reproductive period. The non-inflammatory disorders of the female genital tract (93.33%, *n* = 28) are most related to the tertiary sector. The distribution in health services was the following: 71.30% (*n* = 305) in the primary sector, 21.70% (*n* = 93) in the secondary sector and 7% (*n* = 30) in the tertiary sector.

**Conclusion:**

The studied women presented non-inflammatory disorders of the female genital tract and diseases of the urinary system as determined by gynecological diagnoses. Low-assistance complexity followed in most cases.

## Background

In Brazil, the health system is organized at levels of attention according to the complexity of care services, seeking to provide universal access, equity and equality [1–4]. The primary level of care represents access and low care complexity, carrying out prevention and health promotion actions. The levels of secondary and tertiary care are responsible for performing diagnoses and specific treatments, as well as providing the primary level of continuing education in service [5–7].

Obstetrician-gynecologists have been specialist physicians in delivering care to women, especially regarding health promotion and high complexity for specific treatments [8, 9]. In the national context, especially in the primary sector, the gynecologist acts as an educating care service for other health professionals (family and community doctors, general pediatricians, and general practitioners) [6, 9].

Demand for gynecologic and obstetric care services is projected to rise from 6 to 10% in developed and developing countries [10, 11], and 50–81% of the time of checkups by obstetrician-gynecologists (OB-GYNs) is currently dedicated to women of reproductive age (18–44 years old) with a growth estimate of 7% in the non-reproductive period [12, 13].

In women’s health, studies that associate gynecological diagnoses and their distribution at different levels of complexity and health services are scarce [13, 14], although they provide benefits in health promotion, medical and interdisciplinary education, as well as rationalization according to level-of-care complexity [15, 16]. The use of ambulatory and hospital care services has limited results in the capacity to describe the search behavior of women in relation to obtaining integral health care [16, 17]; the gynecological concerns most often cited are menstrual disorders, other forms of normal bleeding of the female genital tract, inflammatory processes, and urogenital dysfunctions [13–16].

The characterization of the assisted population in the different levels of health services contributes to the quality of health care [17], which results in topics pertinent to women’s health in outpatient interdisciplinary training [18, 19] and fundamental points in the hierarchy of health services in which promotion and treatment measures are still incipient.

Thus, the objective is to analyze the clinical-demographic characteristics, main diagnoses in gynecological ambulatory settings, and their distribution in health services.

## Method

### Study design and sample study

This is a research method of retrospective audit study design with chart review of women who attended the Women’s Health Ambulatory Clinic of the University Hospital of the University of São Paulo, São Paulo, Brazil, from 2012 to 2014.

The research idea was to study clinical observation applications in quality improvement and practice audits from the teaching and learning of service gynecology for Family and Community Medical Residents. This service receives female patients with unsatisfactory or unexpected clinical management from seven Basic Health Units at Unify Health System - São Paulo, Brazil.

Data were selected from a convenience sample of 428 charts from the first medical visit. We calculated the power of the sample test, considering a 95% confidence level, a desired maximum error of 5%, and a population of women over 15 years old [20] who visit the gynecology outpatient clinic each year, with an expected sample size of 227 cases.

### Data source

Data were collected from medical records of the patients. The features were static data with demographic, medical history, and snapshots in time information. The data were feasible, acceptable and reliable (consistent). The standardizing data abstraction tool had responses for categorical variables specify single responses, multiple responses or coded responses. All the responses were collected in Excel.

All the information regarding clinical-demographic and gynecological profiles, type of treatment, and medical referrals were extracted from standardized gynecologic medical records, and a database in Excel format was created. Data were checked regarding information consistency after data collection, and the medical records were re-read when discrepancies were present.

### Data abstraction tool

Clinical and demographic characteristics and non-oncological gynecological diagnoses initially were described. Next, the type of treatment received by the patients in clinical and surgical care was identified. The proportions among clinical diagnoses, reproductive status (presence of menstrual cycles), and non-reproductive status (absence of menstrual cycles greater than 12 months and hysterectomized patients above 40 years) were considered. The variables were grouped by demographic characteristics (age, ethnicity, profession) and clinical characteristics (main complaint, clinical and gynecological diagnosis, concomitant diseases multimorbidity, age of menarche and menopause, onset of sexual activity, parity and smoking).

Data from the gynecological diagnoses collected were standardized according to the International Classification of Diseases in its tenth revision [21]. We grouped the principal diagnoses in five disease categories [14]: 1) Diseases of the urinary system (N30 - N39) – urinary incontinence, cystitis, neuromuscular disorders of the bladder, other disorders of the urinary system, urethritis and urethral syndrome, urethral stricture, other urethral disorders, bladder disorders; 2) Disorders of the breast (N60 - N64) - benign mammary dysplasias, inflammatory disorders of breast, hypertrophy of breast, unspecified lump in breast, fistula and fissure of the nipple, fatty necrosis of the breast, atrophy of breast, non-associated to birth galactorrhoea, mastodynia, solitary cyst of the breast; 3) Inflammatory diseases of female pelvic organs (N70 - N77) - lower genital tract infections (herpes, gonorrhea, chlamydia, Trichomonas, Candida, vulvovaginitis, syphilis), infectious vulvar lesions, inflammatory disease of the upper genital tract, such as disease of the uterus, ovaries, fallopian tubes including cervicitis, salpingitis, endometritis, and tube-ovarian abscess, diseases of Bartholin gland, vulvovaginal ulceration and inflammation; 4) Non-inflammatory disorders of the female genital tract (N80 - N99) – genital dysplasia (precancerous lesions of the vulva, vagina, and cervix), menopausal disorders, menstrual disorders and hormonal dysfunction (dysfunctional uterine bleeding, ovarian hyperestrogenism, ovarian dysfunction, or irregular menstrual Cycles), endometriosis, malignancy of the reproductive tract (carcinoma in situ and invasive disease of the genital tract) benign disorders of the uterus and ovaries (benign ovarian cysts or tumors, leiomyomas, endometrialpolyps, orhyperplasia), infertility; 5) General physical examination, contraception and procreation (Z00 - Z31) – general examination and investigation of people without complaints about contraception, general advice about contraception, insertion of contraceptive devices (intrauterine), sterilization and measures of procreation [10, 11].

The group was based on similarities in clinical symptoms and patient presentation and similarities in management or diagnostic assessment. The diagnostic categories were created by one of the authors and reviewed by two clinical gynecologists. In cases where a single patient presented two or more diagnoses, each of these was described separately. We excluded patients diagnosed with pregnancy, childbirth, puerperium, and cancer, as well as those whose information was incomplete. We excluded ectopic pregnancy because a major proportion of these cases are managed in the hospital rather than in an ambulatory clinic.

### Site of care

Health services were characterized by the type of provided care; this analysis focused on three different sectors: primary sector (basic health unit and school health center), secondary sector (university hospital, hospital of medium complexity, and specialty ambulatory clinics), and tertiary sector (hospital of high complexity and hospital with cancer support). The distribution of health services was based on the sector of origin (referencing) and the sector of destination, where the patient received care or returned to the place of origin (counter-referencing).

### Statistical analysis

In the statistical analysis, the grouping of variables was performed, and these variables were described in tables, as well as measures of central tendency and dispersion. We used the frequency of occurrence of the health diagnoses in gynecology whether categorized among grouped women in the reproductive period or not. The Chi-square test for qualitative variables, Crude Odds Ratio, and 95% confidence interval were performed to verify the difference between the frequency of the categories and the reproductive periods. The Chi-square test was used for qualitative variables and Crude Relative Risk for the proportion of gynecology diagnosis in relation to the distribution of health sectors. A *p* value <0.05 was considered to be statistically significant. When the categories of the variables were analyzed, there were fields that were not filled. However, in order to generate reliable data from the experimental empirical basis, they were transformed into a “missing” category.

This research project was analyzed and approved by the Ethics Committee of the Medical School of the University of São Paulo, according to protocol no.228/13. The ethics committee waived the need for informed consent by participants in the current study.

## Results

Data were collected from the 428 medical records of women at their first visit according to clinical and gynecological factors; women were aged between 13 and 94 years old, with an average age of 45.7 and a standard deviation (SD) of ±14.3 years old, as presented in Table 1.

**Table 1:** Clinical-demographic characteristics, gynecological and obstetric history of patients seen at the Women’s Health Ambulatory Clinic of the University Hospital at São Paulo University, São Paulo, Brazil (2012–2014)

Characteristics	Mean ± Standard deviation	N	%
Age group (years old)	45.7 ± 14.3		
<35		103	24.07
35–44		112	26.17
45–54		100	23.36
55–64		68	15.89
65–74		37	8.64
>74		8	1.87
Ethnicity			
White		251	56.64
Non-White		177	41.36
Profession			
Employed		236	57.42
Non-Employed		175	42.58
Smoking			
Yes		71	20.52
No		275	79.48
Multimorbidity (> = 2)			
Yes		72	17.78
No		333	82.22
Age at menopause (years old)	49 ± 6.0		
<40		11	8.15
40–45		26	19.26
46–54		70	51.85
≥ 55		28	20.74
Coitarche	18.3 ± 8.7		
< 15		32	10.13
15–20		221	69.94
> 20		63	19.94
Active Sexual Life			
Yes		253	70.47
No		106	29.53
Parity			
Nulliparous		70	17.11
1–2		152	37.16
≥ 3		187	45.72
Menarche Age (years old)	13 ± 2.2		
8–12		160	44.08
13–16		181	49.86
> 16		22	6.06

The average age of menarche was 13.0 years old (sd ± 2.20), onset of sexual activity was 18.3 years old (sd ± 8.70), and menopause was 49.0 years old (sd ± 6.0). Concerning sexual activity, 70.47% (*n* = 253) had an active sexual life. Regarding parity, 45.72% (*n* = 187) had three or more children. In relation to the presence of concomitant diseases, 17.78% (*n* = 72) had two or more associated morbidities, and 79.48% (*n* = 275) did not smoke. When demographic factors were analyzed, it was verified that 56.64% (*n* = 251) were White, and in relation to remunerate activity, 57.42% (*n* = 236) were economically active.

In Table 2, the main health diagnoses for non-oncological gynecology, considering both reproductive and non-reproductive periods, were the non-inflammatory disorders of the female genital tract (81.07%, *n* = 347) and diseases of the urinary system (22.66%, *n* = 97).

**Table 2:** Proportion of gynecology health diagnoses regarding the reproductive and non-reproductive periods of women seen at the Women’s Health Ambulatory Clinic of the University Hospital at São Paulo University, São Paulo, Brazil (2012–2014)

Types of Diagnosis (ICD-10)	Reproductive period		Non-reproductive period			
	N	%	N	%	p*	OR** (95% CI)
Diseases of urinary system (N30-N39)	44	13.33	53	29.78	reference	1.00
Disorders of breast (N60-N64)	12	3.64	4	2.25	0.03	3.61 (1.00; 16.29)
Inflammatory diseases of female pelvic organs (N70-N77)	22	6.67	9	5.06	0.01	2.94 (1.15; 7.98)
Non-inflammatory disorders of female genital tract (N80-N99)	236	71.52	111	62.36	<0.001	2.56 (1.58; 4.16)
General physical examination, contraception and procreation (Z00–31)	16	4.85	1	0.56	<0.001	19.27 (2.73; +∞)
Total	330	100	178	100	–	–

*Chi-square test for qualitative variables

**Crude Odds Ratio, 95% confidence interval

In addition, the chances of having disorders of the breast and non-inflammatory disorders of the female genital tract during the reproductive period corresponds to being 3.61 (CI 1.00–16.29) and 2.56 times (CI 1.00–4.16) higher, respectively, than the chance of having the disease in the non-reproductive period (*p* < 0.001).

The non-inflammatory disorders of the female genital tract were 71.52% (*n* = 236); diseases of the urinary system were 13.33% (*n* = 44); inflammatory diseases of female pelvic organs were 6.67% (*n* = 22); general physical examination, contraception and procreation were 4.85% (*n* = 16) and diseases of the breast were 3.64% (*n* = 12); these are the most frequent non-oncological gynecological diagnoses during the reproductive period in the visits of these women. There were a higher proportion of non-inflammatory disorders of the female genital tract (62.36%, *n* = 111) and diseases of the urinary system (29.78%, *n* = 53) in the non-reproductive period.

The chances of diagnosis of non-inflammatory disorders of the female genital tract and general physical examination, contraception and procreation are significantly different (*p* < 0.05) during the non- reproductive period.

**Fig. 1:**
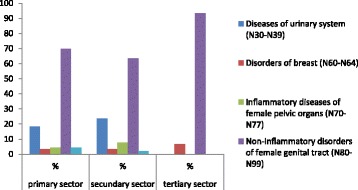
Proportion of gynecology diagnoses in relation to the distribution of health sectors at Women’s Health Ambulatory Clinic of the University Hospital at São Paulo University, São Paulo -Brazil (2012–2014)

With respect to the relationship between the main health diagnoses in gynecology and the distribution of health services (Table 3 and Fig. 1), the non-oncological gynecological diagnoses mostly referred to the tertiary sector and the non-inflammatory disorders of the female genital tract (93.3%). Diseases of the urinary system, general physical examination, contraception and procreation are health diagnoses that remain in the primary and secondary sectors in the distribution of health services.

**Table 3:** Proportion of gynecology diagnoses in relation to the distribution of health sectors at Women’s Health Ambulatory Clinic of the University Hospital at São Paulo University, São Paulo, Brazil (2012–2014)

Types of Diagnosis	Primary			Secondary			Tertiary**		
	N	%	p*	N	%	p*	N	%	p*
Diseases of urinary system (N30-N39)	56	18.36		22	23.66		–	–	
Disorders of breast (N60-N64)	10	3.28		3	3.23		2	6.67	
Inflammatory diseases of female pelvic organs (N70-N77)	13	4.26		7	7.53		–	–	
Non-inflammatory disorders of female genital tract (N80-N99)	213	69.84		59	63.44		28	93.33	
General physical examination, contraception and procreation (Z00–31)	13	4.26		2	2.15		–	–	
			0.68			0.25			0.02
Total	305	100		111	100		31	100	

*Chi-square test for qualitative variables

**Crude Relative Risk (RR) for *p* < 0.05

Considering *p* < 0.05 the crude relative risk was calculated

RR = 5.97 (1.45: 24.70) [Non-inflammatory disorders of female genital tract (N80-N99)];

RR = 1.97 (0.52: 7.50) *p* = 0.33 [Disorders of breast (N60-N64)]

**Fig. 2:**
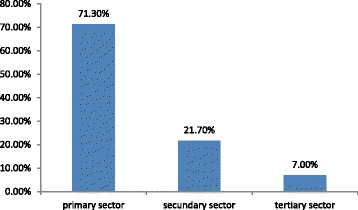
Proportion of the number of patients in relation to the distribution of health services to the sector of destination

With regard to the destination of the patients, following the distribution of health services utilization since the Women’s Health Ambulatory Clinic of the University Hospital at São Paulo University (Fig. 2), 71.2% (*n* = 305) returned to the primary sector, 21.7% (*n* = 93) remained in the secondary sector, and 7.0% (n = 30) were referred to the tertiary sector.

## Discussion

The studied women in this research are economically productive. Regarding clinical-gynecologic characteristics, they are in their late reproductive period, are sexually active, have formed offspring (multiparity), have at least one associated morbidity and are non-smokers. The main non-oncological gynecological diagnoses were non-inflammatory disorders of the female genital tract and diseases of the urinary system both in the reproductive and non-reproductive periods. With respect to the final destination of patients following the distribution of health services utilization since the referral ambulatory clinic in gynecology, it is highlighted that they return to the primary health care sector.

The gynecological and obstetric histories found that the patients resemble those described by other authors in the national territory, such as menarche and menopausal ages [22–24] and the presence of sexual dysfunctions [25], with no variation among other studies performed in developed countries [26].

The traditional health system information and care provided are limited due to lack of knowledge of the characteristics of those who do not seek health services. Therefore, population-based prevalence studies, when representative, have an advantage and allow estimates of epidemiological behavior for the entire community in which they were performed [16]. Considering the use of outpatient health services as a condition that demonstrated access to the specialized outpatient service through the basic network, as in our study, it revealed the main diagnoses in women’s health that result from these services and contributes to the planning and programming of health activities that better reflect reality.

Further evaluation of the connection between changes in the health system and the observed trends and guidelines could not be assessed given that several different guidelines on women’s health screening were issued in our country [27, 28].

Mustard et al. (1998) highlighted the importance to provide an empirical context for the ongoing investigation of equity in the distribution of health care [29]. Nicholson et al. (2001) in a national cross-sectional study in the USA concluded that specific gynecologic diagnoses are associated with the use of emergency departments or hospital outpatient. Instead, descriptive studies of the use of health care services typically document higher per capita use by women during the adult reproductive period [14, 30–32].

Nedel et al. (2008) described that the admission conditions of primary care and the main diagnosis of hospitalization are due to one of the following conditions: being female, schooling, health unit operating time, residing in a family health area and using free care, and medical consultation using research and inpatient hospitalization [33].

The importance of knowledge about levels of the health system and diagnoses improve support for the unified health system in Brazil, whereas the decrease in the tertiary level is consistent with improvement in primary health care [34].The future of the unified health system in Brazil, its sustained expansion to the remaining urban centers, and its effective integration into secondary and tertiary care will require continued engagement by health care providers and the public and continued financial, technical, and intellectual investments [35].

Efforts to ensure that women receive quality care at different levels of health care are performed by care models. Rodrigues et al. (2016) [34] in Colombia reported that changes in the structure of preventive care provided, as well as recommended, specialized services and had a beneficial and significant effect on the performance of women’s health prevention programs offered by primary health care centers. Simon and Uddin (2017) affirmed that the percentage of women who visit specialized services has decreased in the last decade and that in order to guarantee high quality and coordinated care, specialized physicians, among others, should offer all possible recommendations regardless of the level of health care [36]. Dias Costa et al. (2000) in a Brazilian study regarding a medical audit on the prenatal care program in the south of the country noted that the use of the epidemiological methods to organize health services is important to the quality of care [37].

The main health diagnoses in gynecology found in our study, as non-inflammatory disorders of the female genital tract and diseases of the urinary system, have negative influences both on the health and quality of life of women in a late and non-reproductive period [38, 39]. These patients are sexually and economically active, which creates psychological and social consequences.

Non-inflammatory disorders of the female genital tract, including abnormal uterine bleeding, have a prevalence of 40–60% in the reproductive period and may worsen in the late reproductive period due to progressive ovarian dysfunction [40, 41]. Symptoms related to changes in the menstrual cycle can lead to anemia [42]. Abnormal uterine bleeding is also a risk factor for acute hemorrhage and cardiovascular disease, which implies morbidity and mortality [43].

The main non-oncological gynecological diagnosis, described as non-inflammatory disorders of the female genital tract, impacts women’s health in both sexual and labor activity, which causes health imbalances on psychological and social concepts [38, 40]. Adequate clinical management of this entity in primary care becomes relevant to avoid aggravations to women’s health [44, 45].

Moreover, the non-inflammatory disorders of the female genital tract were the non-oncological gynecological health diagnosis most referred to in the tertiary sector, demonstrating [46, 47] the financial impact on the health system. Thus, the clinical control of these patients through the incorporation of drug therapy, which can be performed in the primary and tertiary sectors, is fundamental to avoid high costs and reduce morbidities.

Complaints regarding diseases of the urinary system, common in gynecology, have negative effects on different aspects of women’s lives and have been assisted by primary and secondary levels in our sample [46, 48].

Health professionals working with women’s health for low complexity issues should be aware of and be trained to provide care to women with non-oncological gynecological diagnoses for adequate clinical management, bringing benefits to reproductive and sexual health [6, 22].

In addition, the qualification of health professionals in relation to these priority themes and opportunities remains limited for health care in interdisciplinary services. Our study was conducted in a training program with an emphasis on women’s health [18, 19], and the results therefore found in the main health diagnoses identified may be themes of continuing education.

Inflammatory diseases of female pelvic organs, disorders of the breast, general physical examination, contraception and procreation were the most frequent gynecological diagnoses in the reproductive period of these patients, corresponding to demands related to the menstrual cycle and sexual activity present in the reproductive period of life [19, 22].

Benign breast diseases are more likely to be diagnosed during the reproductive period, but their clinical management was mostly performed in medium- and high-complexity healthcare, demonstrated in our study with presence in the secondary and tertiary sectors. This may corroborate with the specificity of this entity and the importance of the medical specialist in the care assistance of this entity [49].

The survey of our patients identifies the main themes that need investments, such as health education for professionals and the stimulation of multiprofessional work. In our study, 66.5% of cases referred to medium complexity received clinical treatment, reinforcing the importance of training health professionals in the treatment and follow-up of the main diagnoses in women’s health. The distribution of the use of health services was considered adequate according to the literature [50] in countries that adopt hierarchical levels of health care and universal access (e.g., England and Canada). The final destination of patients mostly to the primary sector is considered satisfactory. However, special attention is given to the need to optimize services and referral flows at different levels of attention to the quality of health care [13, 14], especially in women’s health.

Descriptive and retrospective studies have their own limitations when data from medical records are analyzed, especially considering the quality of sociodemographic information recording and in determination of racial classification in Brazil, reflecting non-homogeneous criteria.

The intensity of health services utilization in women’s care may not represent the demand in the unified health system, since it addresses an ambulatory clinic accredited for a teaching and learning facility unit.

The novelty of this study correlates the main diagnoses in women’s health and the hierarchy of the health services levels, associating the demands brought by women and the need of health that is addressed in the different levels of health care. This fact brings benefits in the field of health promotion in women’s health, in the field of medical and interdisciplinary teaching highlighting current issues in the daily life of women, and in public health enabling this ongoing source for health care complexity.

## Conclusion

In conclusion, the attended and referenced women are in the late-reproductive period. The major health diagnoses in non-oncologic gynecology are non-inflammatory disorders of the female genital tract and diseases of the urinary tract. The return to low assistance complexity was present in most cases regarding the distribution of the utilization of services.

## Abbreviations

OB-GYNs: Obstetrician-gynecologists
